# Polymorphisms in the hypervariable control region of the mitochondrial DNA differentiate BPH populations

**DOI:** 10.3389/finsc.2022.987718

**Published:** 2022-11-02

**Authors:** Rashi Anand, S. Priyokumar Singh, Nihar Sahu, Y. Tunginba Singh, Sudeshna Mazumdar-Leighton, J. S. Bentur, Suresh Nair

**Affiliations:** ^1^ Plant-Insect Interaction Group, International Centre for Genetic Engineering and Biotechnology, Aruna Asaf Ali Marg, New Delhi, India; ^2^ Plant Biotic Interaction Lab, Department of Botany, University of Delhi, Delhi, India; ^3^ Department of Botany, Mizoram University, Aizawl, Mizoram, India; ^4^ Agri Biotech Foundation, Hyderabad, India

**Keywords:** *Nilaparvata lugens*, rice pest, genomic Simple Sequence Repeats (gSSRs), *in silico* Restriction Fragment Length Polymorphism (dRFLP), genetic diversity, Insect Pest Management, tandem repeats

## Abstract

The brown planthopper (BPH; *Nilaparvata lugens*) is one of India’s most destructive pests of rice. BPH, a monophagous migratory insect, reported from all major rice-growing ecosystems of the country, is capable of traversing large distances and causing massive crop loss. A crucial step for developing viable management strategies is understanding its population dynamics. Very few reliable markers are currently available to screen BPH populations for their diversity. In the current investigation, we developed a combinatorial approach using the polymorphism present within the mitochondrial Control Region of BPH and in the nuclear genome (genomic simple sequence repeats; gSSRs) to unravel the diversity present in BPH populations collected from various rice-growing regions of India. Using two specific primer pairs, the complete Control Region (1112 to 2612 bp) was PCR amplified as two overlapping fragments, cloned and sequenced from BPH individuals representing nine different populations. Results revealed extensive polymorphism within this region due to a variable number of tandem repeats. The three selected gSSR markers also exhibited population-specific amplification patterns. Overall genetic diversity between the nine populations was high (>5%). Further, *in silico* double-digestion of the consensus sequences of the Control Region, with HpyCH4IV and Tsp45I restriction enzymes, revealed unique restriction fragment length polymorphisms (digital-RFLPs; dRFLPs) that differentiated all the nine BPH populations. To the best of our knowledge, this is the first report of markers developed from the Control Region of the BPH mitogenome that can differentiate populations. Eventually, such reliable and rapid marker-based identification of BPH populations will pave the way for an efficient pest management strategy.

## Introduction

Rice is the staple food consumed by more than half of the world’s population, particularly in Asia, Latin America, and Africa (1). A substantial economic loss in crop productivity due to insect pests is predicted, especially in a warming climate (2). Yield losses up to 30% due to pests alone have been reported for rice in a recent survey (3). In Asia, the brown planthopper (BPH; *Nilaparvata lugens*; Hemiptera; Delphacidae) is the number one pest of rice and is estimated to cause an annual crop yield loss worth more than US$ one billion in China alone (4).

BPH is a small, ochraceous brown, phloem sap-sucking monophagous insect. It exhibits density-dependent wing-dimorphism (5). The macropterous insect is more suited for flight and long-distance migration, while the brachypterous form is highly fecund, resulting in exponential population growth. Also, because of its monophagous feeding habit, the macropterous forms traverse thousands of kilometers, searching for their host (6). Extensive BPH infestation in rice fields results in wilting and browning of the plant and eventually plant death, a condition known as hopper burn (7, 8). This insect is ubiquitously present in all the major rice-growing areas of the country (9).

During the last three decades, with frequent pest outbreaks across Asia, this pest has re-emerged as a significant threat to rice production (10). The rapid breakdown of host resistance and the emergence of pesticide resistance in BPH populations are serious concerns. One of the main causes of these pestilent outbreaks is the deployment of broad-spectrum generic chemical pesticides along with fertilizer-intensive agriculture (11). Therefore, to minimize the destruction caused by this pest and devise efficient and environment-friendly strategies for its control and management, it is vital to understand the population structure of BPH. Specifically, keeping in mind the migratory nature of this insect, it is essential to understand their location-specific population features, i.e., origin, lineage, distribution, movement patterns, and population dynamics, for developing effective pest management practices.

Understanding the population structure of migratory insect pests is essential to identify the origin and path of migration, the evolutionary history of local populations, gene flow, and the spread of insecticide resistance or virulence among the populations (12–19). In this regard, DNA-based molecular markers would be an ideal tool for the realization of these objectives. Different molecular markers provide genetic information best suited for specific phylogenetic studies. With the advent of reliable DNA-based molecular markers, various such markers are being used for studying insect populations. DNA markers such as RAPDs (Random amplified polymorphic DNA), AFLP (Amplified fragment length polymorphism), mitochondrial DNA, ESTs (Expressed Sequence Tags), and microsatellites have been previously used to analyze insect phylogeny (20, 21). RAPD markers, however, have been reported to show non-reproducible or unreliable results in many insects such as gypsy moth (*Lymantria dispar* Linnaeus) and BPH (20, 22, 23). Though AFLP markers are highly reproducible, they cannot be employed as a reliable marker system for phylogenetic analyses due to their dominant nature. Of the several DNA markers, those developed from the mitogenome of insects have been successfully utilized for various phylogenetic studies to identify the origin of species, phylogeographic analysis, and studies involving population dynamics (24, 25). Currently, the analysis of mitochondrial DNA is becoming a powerful tool for assessing the genetic relatedness of species.

Mitochondrial DNA-based markers are most often used in such studies (26, 27). Specifically, polymorphism in the cytochrome oxidase 1 (*cox1*) mitochondrial gene sequence has been shown to be useful for DNA barcoding to distinguish insect species (28). *cox1*-based markers are also used in characterizing invasive and migratory insect populations (29). However, such markers are not free from limitations (24, 30). Other mitochondrial genes targeted for similar studies are *nad1* (NADH dehydrogenase 1) (29) and *cox2* (31).

The mitogenomes of all the three rice planthoppers, BPH, White backed planthopper (WBPH; *Sogatella furcifera*) and Small brown planthopper (SBPH; *Laodelphax striatellus*), have been sequenced (32–36). Variations in the mitogenomes of SBPH populations of China and Japan are reported and linked to evolutionary diversity (37). Various studies have elucidated the genetic divergence and identified migration routes and sources of immigrating populations of BPH in various South-Asian countries using mitochondrial genes such as *cox1, trnL2 and cox2* and through whole-genome sequencing (38–41). However, based on partial sequence information on the mitochondrial cox1 gene, Srinivasa et al. (42) concluded that geographically distinct BPH populations do not exist in India.

It is evident from the above reports that variations among BPH populations within India are not discernible. However, variations in response to rice varieties carrying different resistance genes or levels of resistance against commonly used insecticides within India have been well documented (43, 44). Therefore, a more accurate, reliable, cost-effective, and rapid marker system is needed to identify and differentiate BPH populations within India. The mitochondrial Control Region can be a basis for the development of a powerful tool for analyzing the variability present within insect populations (25, 45). Although it is a non-coding region, it controls DNA replication and regulates the transcription of the mitochondria in the cell (46). As a result of the variable copy number of tandem repeats present in this region, previous studies have indicated length polymorphisms in the mitochondrial genome (32). Such polymorphisms also contribute to the size variations observed among various inter-cellular and intra-cellular mitogenomes (47). Based on these observed intraspecific size and length variations in the BPH mitogenome, the current investigation focused on analyzing these polymorphisms in the Control Region of the BPH mitogenome to develop a reliable molecular marker system for differentiating BPH populations collected from various rice-growing areas in India.

In the current study, using a combinatorial approach, we analyzed the number of tandem repeats present in this region, and along with SNP analysis and variations in restriction patterns (obtained after *in silico* restriction fragment length polymorphism (RFLP) analysis), we explored the genetic variability present among BPH populations collected from geographically distinct rice-growing ecosystems. Additionally, to further enhance this study’s effectiveness, reliability, and robustness, we screened a few nuclear-gSSR markers identified in an earlier study (21) for assessing genetic diversity in BPH.

To the best of our knowledge, this is the first study highlighting the polymorphisms present in the Control Region of the mitogenome of BPH. Results obtained will help understand and correlate genomic variations and population differentiation. Keeping these objectives in focus, the present investigation was carried out targeting the tandem repeats in the Control Region of the mitogenome to develop markers and use these along with nuclear genomic SSR markers to differentiate Indian populations of BPH.

## Materials and methods

### Collection, DNA isolation and whole genome amplification of BPH

BPH adults from nine different locations, as shown in **Figure S1**, Cuttack (20°28’N 85°53’E), Odisha; New Delhi (28°31’N 77°10’E), Delhi; Hyderabad (17°19’N 78°24’E), Telangana; Bishnupur (24°39’N 93°52’E), Manipur; Aizawl (23°43’N 92°43’E), Mizoram; Nalgonda (17°04’N 79°15’E), Telangana; Ludhiana (30°54’N 75°51’E), Punjab; Rajnagar (24°02’N 91°39’E), Tripura; and Warangal (17°58’N 79°35’E) Telangana, were collected from the rice fields and stored in absolute ethanol at -20°C. Before DNA isolation, individual insects were de-winged and crushed in liquid nitrogen using sterile micropestles. Following the manufacturer’s protocol, the DNA was isolated from individual insects constituting the different populations using the GF-1 Tissue DNA Extraction kit (Vivantis Technologies, Malaysia). The DNA was eluted in preheated sterile MilliQ water. The DNA was quantified spectrophotometrically using NanoVue (GE Healthcare, UK) spectrophotometer. As the amount of DNA obtained from individual insects was insufficient to carry out all the envisaged downstream experiments, we resorted to whole genome amplification (WGA) to increase the amount of available DNA for each BPH DNA sample. WGA was carried out using the V2 Genomiphi Whole Genome Amplification kit (GE Healthcare, UK), following the manufacturer’s instructions. The genomic DNA obtained was quantified spectrophotometrically. All the analysis reported in this investigation was carried out using the whole genome amplified DNA.

### PCR amplification, cloning and sequencing of the mitochondrial Control Region of BPH

**Figure 1:**
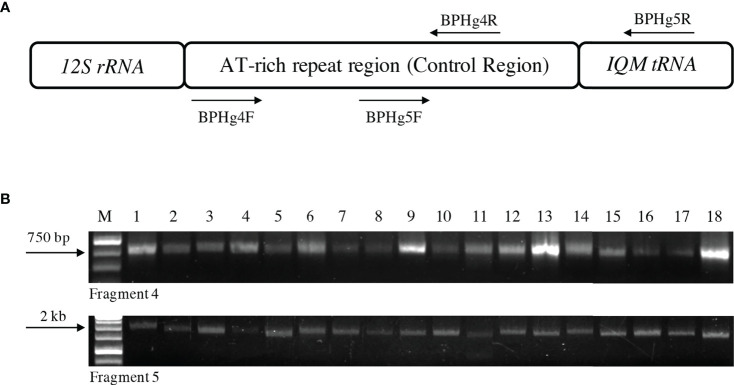
The Control Region of the mitochondrial genome of *Nilaparvata lugens*. **(A)** Diagrammatic representation of the location of the Control Region in the BPH mitochondrial genome indicating mitochondrial genes flanking it. Arrows indicate the two PCR primer pairs (BPHg4F and 4R and BPHg5F and 5R) used for the PCR amplification of the entire Control Region in two overlapping fragments. **(B)** A representative agarose gel (1%) depicting heterogeneity in the PCR amplified fragments of the Control Region from two individuals in each of the nine populations (Lanes 1 to 18). The upper and lower panels represent fragment 4 (~780 bp) and fragment 5 (~1.8 kb), respectively. M: 1 kb molecular weight ladder; Lanes 1-18. Two individuals each of Cuttack, New Delhi, Hyderabad, Nalgonda, Ludhiana, Bishnupur, Aizawl, Rajnagar and Warangal populations, respectively. The arrows on the left represent molecular weights as indicated.

The entire Control Region of the BPH mitogenome was PCR amplified using two primer pairs, BPHg4F/R and BPHg5F/R (32) (**Table S1**), as two overlapping fragments, i.e., fragment 4 and fragment 5 (**Figure 1**). The two fragments were generated using the following PCR conditions: an initial denaturation at 95°C for 30 sec; followed by 30 cycles at 95°C for 30 sec, annealing at 45-47°C (for fragment 4) or 55-57°C (for fragment 5) for 30 sec, extension at 65°C (for 30 sec for fragment 4 and 60 sec for fragment 5); and a final extension at 65°C (for 30 sec for fragment 4 and 60 sec for fragment 5). Annealing and extension temperature for the PCR amplification of both fragments varied and was standardized for each population. Each PCR reaction (25 μl) contained 200 μM dNTPs, 1× Taq buffer, 0.5 U Taq DNA polymerase (KAPA 2G Robust; Kapa Biosystems, Germany), and ~18 μM of each primer. The PCR products were visualized on a 1.0% agarose gel run in TBE buffer. The PCR products were gel-purified using GF-1 AmbiClean Kit (Vivantis Technologies, Malaysia). The eluted products were cloned into the pCR4-TOPO TA cloning vector using the TOPO-TA cloning kit (Invitrogen, USA) and transformed into *E. coli* DH5α competent cells. Positive clones were selected on LB agar (with Kanamycin 50 µg/ml). Positive colonies from the plates were re-screened using PCR with T3-T7 primer pair (**Table S1**) to ensure the authenticity of the size of the cloned inserts. The constituents of the PCR mix were as described before, except that Taq polymerase was used (Bangalore Genei) 1.2 U along with its respective 1X Taq Buffer in a 20 μl PCR reaction. As described earlier, the PCR products were electrophoresed on a 0.8% agarose gel. Two PCR-positive clones representing each of the two fragments from all individual insects screened in this study were selected for all the populations. The plasmid DNA was isolated from each clone selected using the Qiaprep Spin Miniprep kit (Qiagen, Germany). Both clones representing each fragment (4 and 5) for all the samples were sequenced using the Big Dye Capillary Sequencing method by M/S Macrogen Inc. (Seoul, South Korea) using M13F and M13R-pUC universal sequencing primers.

### Nucleotide sequence analyses

The raw sequencing data obtained for each clone was subjected to Phred, Crossmatch and Phrap analyses for base calling, vector trimming and contig assembly, respectively, using the MacVector suite of sequence analysis programs (version 16.0.8, MacVector Inc., USA). Using Phrap and sequences of fragments 4 and 5, the entire Control Region of the BPH mitogenome was assembled for every individual screened and as represented in **Figure 1**. Using an online Tandem Repeats Finder tool (48), the nucleotide sequences of the entire Control Region of all the samples were scanned for the presence of tandem repeats. The copy number of the repetitive elements present in the Control Region was recorded.

### PCR Amplification and analysis of the genomic simple sequence repeats (gSSRs)

Genomic SSRs were also analyzed using the WGA DNA of the BPH individuals collected from different geographical locations. The primers used for PCR amplification of these microsatellite markers were those used earlier by Jing et al. (21). Various primer pairs were tested and screened at PCR conditions specific to each pair. Subsequently, three gSSRs were shortlisted for this study (**Table S1**). 20 μl of each PCR reaction was set as mentioned earlier, using Bangalore Genei Taq DNA Polymerase. PCR conditions included an initial denaturation at 95°C for 120 sec; followed by 30 cycles of denaturation at 95°C for 30 sec, annealing 50°-55°C (depending on the primer pair used; **Table S1**) for 30 sec, extension at 72°C for 45 sec; and a final extension at 72°C for 120 sec. The PCR amplified products were electrophoresed on a 2.5% agarose gel and the gels were photographed using a gel doc system (AlphaImager HP Imaging system, Protein Simple; USA) and the gel pictures were analyzed using Image Lab software (version 6.0, Bio-Rad Laboratories, Inc.). The gSSR markers that were able to differentiate the various populations (based on their amplified length polymorphisms) were selected and used for further analysis.

### Statistical analyses

The number of repeat motifs identified by Tandem Repeat Finder in each fragment and the molecular weights of the PCR amplified gSSR marker fragments were further analyzed using MetaboAnalyst (http://www.metaboanalyst.ca/). The raw data were normalized by sum and log-transformed (natural log). The data were also auto-scaled (mean-centered and divided by the standard deviation of each variable) computationally before the Kruskal-Wallis test and PLS-DA (Partial Least Score-Discriminant Analysis) were conducted. VIP (Variable Importance in Projection) coefficients and scores were retrieved from PLS-DA results. The top 5 markers with maximum scores obtained after the Kruskal-Wallis test and high VIP scores were used to carry out PCA (Principal Component Analysis) using an online tool ClustVis (https://biit.cs.ut.ee/clustvis/). Mean score values for each population were obtained using default parameters.

### Multiple sequence alignment (MSA) and single nucleotide polymorphism (SNP) analyses

CodonCode Aligner (CodonCode Corporation, USA) was used to carry out MSA of the sequences obtained for the Control Region for all the BPH individuals screened. All samples of each population were aligned using MUSCLE built into the CodonCode Aligner software end-to-end alignment algorithm with a minimum of 80% identity. A consensus sequence was generated for each population based on this alignment. Next, the MSA of nine consensus sequences was carried out using the same algorithm. All the observed SNPs between the consensus sequence, specific for each population, were recorded and tabulated based on the alignment. Next, the consensus sequences of these populations were subjected to *in silico* restriction analyses utilizing more than 200 restriction enzymes (digital RFLP) using the MacVector suite of programs. The program generated an agarose gel simulation to easily visualize the restriction pattern generated for each restriction enzyme tested against the consensus sequences of the Control Region of the BPH mitogenome across all populations screened. The restriction enzymes that could provide reliable and distinguishable results were used for further analysis. A phylogenetic tree was also constructed using MacVector suite of programs. The aligned consensus sequences of the complete control region of all nine populations were used for building the phylogenetic tree using UPGMA (Unweighted Pair Group Method with Arithmetic mean) method. The bootstrap consensus tree was inferred from 1000 replications to represent the genetic relationship of the populations analyzed. The evolutionary distances were computed using Tamura-Nei method within MacVector suite of programs.

### Analysis of genetic diversity

The genetic relatedness between the BPH populations was quantified by analyzing the variations at the nucleotide level in the Control Region. Using sequence data, inter and intra-population nucleotide diversity was calculated using classic theories of genetic diversity (heterozygosity), defined within a population context, i.e., Nei & Li’s nucleotide diversity see (49) by estimating ‘π’.


$$\pi =\left(n/n-1\right)*\text{Σ}X_{a}X_{b}\pi_{ab}$$

where, π = Degree of nucleotide diversity present in the entire population (intra- or inter-)

n = Number of samples in a population or populations under analysis

X_a_ = Estimated frequency of the sequence of sample ‘a’ (in case of intra-population) or population ‘a’ (in case of interpopulation)

X_b_ = Estimated frequency of the sequence of sample ‘b’ (in case of intra-population) or population ‘b’ (in case of interpopulation)

π_ab_ = Proportion of SNPs between sequence of sample ‘a’ and ‘b’ or population ‘a’ and ‘b’

π was calculated for all nine populations by calculating the pairwise proportions of SNPs and estimated frequencies of the sequence of the samples. The degree of genetic diversity between these nine populations was calculated, and a similarity matrix was generated by calculating pairwise π. Intra-population nucleotide diversity was also calculated. Additionally, nucleotide diversity was observed and calculated between populations representing geographical groups such as north, south and north-east regions.

## Results

The complete Control Region of individuals sampled (N=53) from nine different BPH populations was PCR amplified, cloned, sequenced and analyzed. The Control Regions were amplified as two overlapping fragments comprising fragment 4 (783 to 815 bp) and fragment 5 (872 to 2316 bp). Subsequently, a contiguous sequence representing the entire Control Region was obtained by joining the overlapping sequences of these fragments. The sequence length of the Control Region thus obtained varied from 1112 to 2612 bp. Sequence analysis of this region identified several sequence characteristics which could differentiate the BPH populations. At this stage, length polymorphism was observed between some of the populations based on the amplification of fragments 4 and 5 of the Control Region (**Figure 1**) due to the variable numbers of tandem repeats present in this region (discussed below in detail). The tandem repeats (TR) analysis revealed the number of iterations and their motifs (**Table S2**) in all the populations. Of the different TR motifs identified, ten repeat motifs were differently iterated, and these were observed and recorded for all the individuals in the nine BPH populations (**Table S3**). TR10, a 21-bp repeat motif GGAAAAAATGTCACGTTTTT(C/T), ubiquitously present in fragment 5 of all the samples, was the most polymorphic. The iterations of this repeat element varied from 4 (in an individual representing the Nalgonda population) to 74.4 (in an individual representing the Bishnupur population) (**Table S3**), resulting in significant amplification length differences when fragment 5 was PCR amplified from all the individuals. The sequences of the complete mitochondrial Control Region of all nine BPH populations were submitted to NCBI and can be accessed using the following accession numbers: MK792351 to MK792395 and OL964031 to OL964038.

In addition to TR markers, 12 gSSR markers were also screened (see Materials and Methods and **Table S1** for details). Of these, we selected three gSSR markers for subsequent screenings based on their capacity to reveal polymorphism between individuals and populations (**Table S4**). The remaining gSSR markers were not further used in this study as they were monomorphic or failed to amplify or amplified multiple non-specific fragments. The amplification patterns obtained using the three selected gSSR markers (BM1350, BM1369, and BM1373) showed a high degree of polymorphism between the samples representing the different BPH populations (**Table S3**). Regions amplified by BM1350, BM1369 and BM1373 were rich in dinucleotide repeat motifs, TC, AG and CT, respectively. In a few samples, we did observe amplification of an additional fragment in addition to the expected fragment. These bands, usually smaller than the expected fragment size, were considered stutter bands and not used for subsequent analysis.

**Figure 2:**
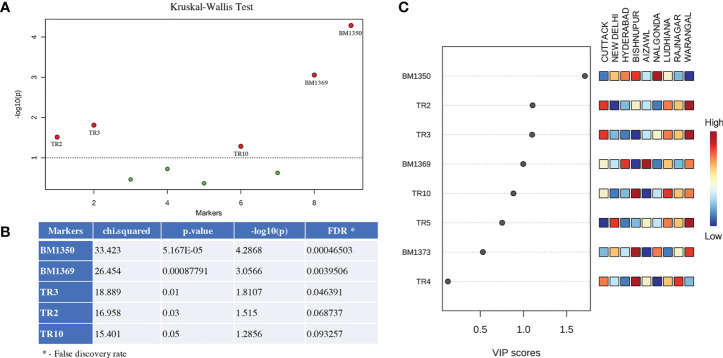
Overview of the statistical significance of the potentially significant markers identified in this study that are capable of distinguishing different BPH populations. **(A)** Significant markers identified by the Kruskal-Wallis test analysis. Markers falling above the threshold (dotted line; *p* ≤ 0.05) indicate robustness of these markers in distinguishing BPH populations. **(B)** Statistical significance of the five most important markers (left column) identified in this study. The chi-squared values and the *p*-value represent the comparisons between markers showing different significance levels at *p*-value threshold of 0.05. **(C)** Variable Importance in Projection plots (VIP) and Partial least square discriminant analysis (PLS-DA) plots calculated for the markers characterized in this study and shown in their descending order of importance. Higher VIP scores indicate a more significant contribution of that particular marker in discriminating the populations. The heat map scores are indicative of the fragment amplified using the SSR markers (names beginning with BM) or the number of iterations in fragment amplified by the tandem repeat markers (names beginning with TR). Marker names are indicated on the left.

The data obtained for the number of tandem repeats present in the Control Region of the mitogenome and the data on gSSR polymorphisms among the BPH individuals were analyzed and subjected to statistical analyses. The Kruskal-Wallis test identified several potential markers capable of distinguishing the different BPH populations studied (**Figures 2A, B**) and the results thus obtained also matched PLS-DA results (**Figure 2C**). Subsequently, data obtained for five markers (TR2, TR3, TR10, BM1369, and BM1350) with high VIP scores (**Figure 2C**) were selected for population differentiation.

**Figure 3:**
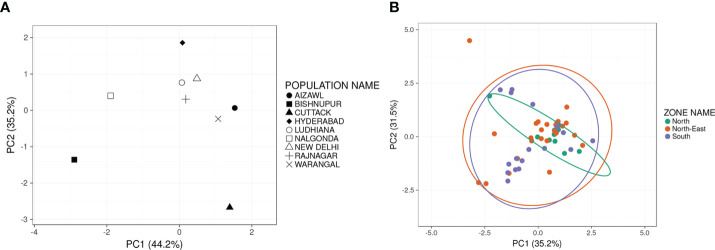
Two-dimensional Principal Component Analysis plots indicating the principal components displaying maximum variance estimated on the basis of marker-based differentiation of populations and grouped on the basis of place of collection **(A)** (N=9) or region of collection **(B)** Each solid circle represents an individual sample of a zonal population. Green solid circles represent individuals of the North populations (Delhi and Punjab); Orange solid circles represent individuals of the North-East populations (Aizawl, Bishnupur and Rajnagar); Purple solid circles represent individuals of the South populations (Cuttack, Hyderabad, Nalgonda and Warangal). Prediction ellipses in **(B)** built with a probability of 0.95 and N=53.

PCA results revealed high variability between the nine BPH populations (**Figure 3**). The top PCs, i.e., PC1 and PC2, reflected and revealed the genetic diversity and heterogeneity among different populations of BPH in India. PC1 and PC2 represented 44.2% and 35.2%, respectively, of the total variation (**Figure 3A**) observed and accounted for >79% of the total variation amongst the nine populations. Next, the PC values of individual samples comprising the nine populations were collapsed based on their mean values such that a single node represented each population (**Figure 3A**). Based on the PCA plot, New Delhi, Ludhiana and Rajnagar and so also Aizawl and Warangal populations were relatively closer. At the same time, Nalgonda and Hyderabad were distant from the central cluster yet their distance from the main cluster was lower than that of Cuttack and Bishnupur. The phylogenetic analysis of the nine populations revealed a similar pattern of genetic divergence among the populations under study (**Figure S2**). When the populations were grouped based on their geographical origin: north, south and north-east, built with probability 0.95 (**Figure 3B**), the two PCs could account for > 66% of the total variance between the three groups.

Further, a detailed *in silico* restriction analysis of the sequences representing the Control Region of these populations provided unique differentiating restriction patterns. These polymorphisms were critical for calculating the overall genetic diversity in these samples and studying inter and intra-population variations. In addition, the consensus sequences thus obtained for all the populations were used to calculate the nucleotide diversity index. Through pairwise comparisons of the consensus nucleotide sequence representing each population, values for the estimated frequency and the proportion of nucleotide present at each variable locus were calculated. Based on these, the overall genetic diversity calculated for the nine populations was 5.8% (**Table S5**).

**Figure 4:**
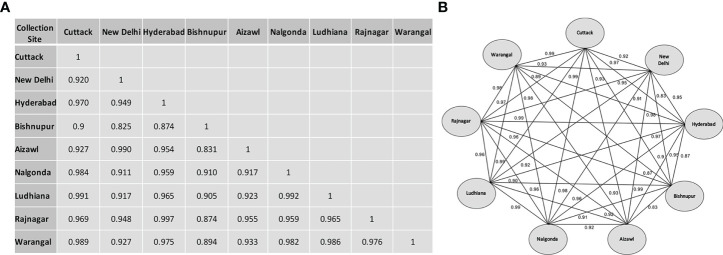
Nucleotide similarity index based on the sequence of the Control Region of BPH individuals collected from different rice-growing regions of India. **(A)** Similarity matrix derived using Nei and Li’s nucleotide diversity index ‘ &’ (see Materials and Methods for details). **(B)** Pairwise relationship network plot illustrating binary comparisons of the nine BPH populations studied. The labels on each connecting line represent the similarity index between the BPH populations.

The inter-population genetic similarity coefficient matrix (**Figure 4A**), derived using Nei and Li’s equation, indicated that despite being geographically distant, Hyderabad and Rajnagar populations showed maximum similarity (0.9972) based on similarity coefficients (**Figure 4B**). Ludhiana-Cuttack, New Delhi-Aizawl, and Nalgonda-Ludhiana also demonstrated exceptionally high similarities. In contrast, populations from New Delhi- Bishnupur and Aizawl-Bishnupur showed maximum variability, with the similarity coefficient estimated to be 0.825 and 0.831, respectively. Bishnupur exhibited maximum diversification compared with the other eight populations due to the high sequence diversity of a 333 bp insertion in their Control Region sequences. In addition, multiple sequence alignment (MSA) revealed several Bishnupur population-specific SNPs, e.g., at nucleotide positions 96, 922, 1718 and 1739 among several others (**Table S6**). This study also analyzed the overall variation among north, south and north-east populations based on the Control Region. The northern population observed 8.3% nucleotide diversity, while the estimated nucleotide diversity was just 2.3% for the southern population. The north-east population showed maximum variation, 11.1%.

The intra-population genetic diversity also clearly indicated that the BPH individuals within a population exhibited high sequence polymorphism. The within-population nucleotide diversities of the nine populations ranged from 3% to 12.2%. Nalgonda and Bishnupur exhibited maximum within-population variation with less than 90% similarity. The MSA analysis identified New Delhi population-specific SNPs at positions 38, 271, 272, 317, 318, and 362 (**Table S6**) in the consensus sequences of the Control Region; however, these nucleotide positions were represented by indels or SNPs. A 20-bp deletion (from nucleotide position 1021 to 1041) was also uniquely present in the individuals constituting the New Delhi population.

Similarly, indels or SNPs at positions 269, 270, 450,451 and 901 were unique to individuals representing the Cuttack population. Individuals representing the Hyderabad population showed unique SNPs at nucleotide positions 985 and 1631, allowing them to be uniquely identified and differentiated from the remaining eight populations. We observed Aizawl population-specific polymorphisms at positions 662, 663, 1713 and 1766. In the Nalgonda population, ‘A’ is conserved at position 1686 in the consensus sequence in contrast to ‘T’ or deletion at that specific position of the other populations. The individuals of the Ludhiana population could be identified by the unique polymorphisms present at positions 1090 and 1195. For the Rajnagar population, ‘A’ is present in place of ‘C’ at positions 1627 and 1648, and in the Warangal population, ‘C’ is present in place of a ‘T’ at position 1022.

**Figure 5:**
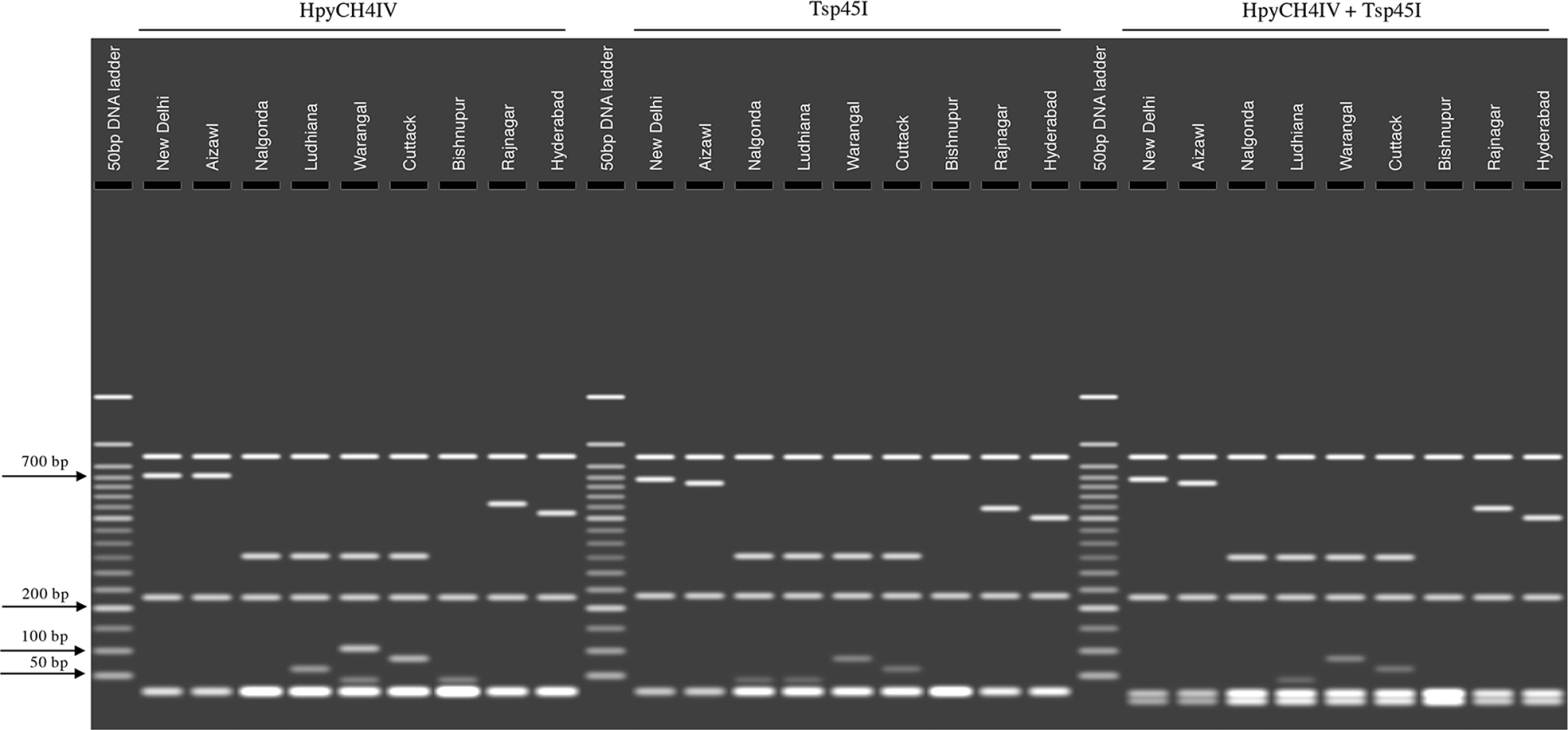
*In silico* visualization of RFLPs between BPH populations based on restriction digestion of the consensus sequence of the Control Region representing each population. The restricted products were visualized on a simulated 1.5% agarose gel (using the MacVector suite of sequence analysis programs (see Materials and Methods for details). The restriction enzymes selected for the simulation were HpyCH4IV and Tsp45I and a double digest combining both these restriction enzymes. BPH population names are indicated above the lanes. Figures on the left indicate molecular weights in base pairs (bp).

To better visualize these unique polymorphisms, we generated a restriction fragment length polymorphism (RFLP) pattern for each population *in silico*. Though several restriction enzyme sites were present in the consensus sequences of each population, restriction enzymes HpyCH4IV and Tsp45I could specifically restrict within the polymorphic regions in the sequences representing each population. Results indicated unique RFLPs for each population (**Figure 5**). Results obtained from the HpyCH4IV digestion showed a discrete restriction pattern for all the populations except those representing New Delhi and Aizawl. Though these two populations share 99% similarity, restriction with the Tsp45I generated an RFLP that differentiated the New Delhi and Aizawl populations. While the two restriction enzymes used singly were unable to distinguish all the nine populations, performing a double-digest using HpyCH4IV and Tsp45I simultaneously revealed nine distinctive RFLP patterns that uniquely identified the nine BPH populations analyzed in the current study (**Figure 5**).

## Discussion

BPH is ubiquitously present in almost all the significant rice-growing areas of India. Moreover, given the fact that rice cultivation occurs around the same time all over the sub-continent, this crop is available as the host for BPH to feed on. However, rice is grown only during the rainy (*Kharif*) season in the northern states, i.e., Delhi and Punjab, in contrast to southern and north-eastern states where two or three crops are grown in a year. Owing to severe winter, BPH does not survive in northern states during the winter season. This implies that there is a likely migration of BPH to reinfest rice in northern states every year to coincide with the rice planting season.

Even though very few reports exist that show population-level differentiation of BPH, it has been reported that BPH populations from various parts of the country respond differently to rice lines carrying different resistance genes (43) and also differ in the level of insecticide resistance (44) and thereby indicating the existence of genetic heterogeneity among the BPH populations within the country. Several BPH resistance genes have been identified (50), and these are usually effective against specific BPH populations (51). Therefore, it is crucial to first acknowledge the differences between populations and then identify the differences harbored by various populations and strategize the management of such pests accordingly.

However, very few reports highlight the genetic composition and diversity among the different BPH populations prevalent in India. Based on sequence information obtained from the mitochondrial cox gene segment amplified by a set of universal barcode primers using 16 individuals and 16 sequences retrieved from the NCBI database, Srinivasa et al. (42) observed high genetic homogeneity among the populations. Based on whole-genome sequence analysis, Hu et al. (40) distinguished five distinct BPH genetic groups across the south, south-east and east Asia and Australia. However, there was no distinction between Indian BPH populations present in the south Asian group. This could be a consequence of the poor resolution of the markers used in these studies. Though mitochondrial cox1 gene sequence is universally used to develop barcodes to distinguish insect species (52), it may not be suitable to distinguish BPH populations. Length polymorphism arising from differences in iterations of simple sequence repeats forms the basis of SSR markers generally targeting non-coding regions of the nuclear genome. Such markers have also been of limited use in studying the population diversity of BPH. Though the AT-rich Control Region of the mitochondrial genome of BPH has been reported to contain tandem repeats (TR) (32), no prior attempts have been made to target this region to develop reliable markers. Length heteroplasmy in the mitochondrial DNA has often been associated with the variable number of tandem repeats in the Control Region of the mitogenome (53). In the current study, we attempted to utilize variations present in the TRs of the Control Region to differentiate BPH populations.

In this study, one of the primary reasons for utilizing these repeats as markers is their capacity to reveal extensive polymorphisms due to very high variation in their iterations (**Table S3**). In addition, we could also improve the resolving capacity of these markers by resorting to *in silico* restriction of these iterated regions with specific restriction enzymes, i.e., HpyCH4IV and Tsp45I. Further, in a combinatorial approach, the polymorphisms revealed by the gSSR markers were also derived from length variability (arising due to variability in the iterations of the repeated elements within them). Results obtained from five such combined markers: TR2, TR3, and TR10, along with BM1350 and BM1369, distinguished all the nine populations tested. gSSRs that are used as genetic markers usually have repeats embedded in the non-coding regions of the genome and are generally presumed to evolve neutrally (54, 55). Moreover, their frequency and ubiquitous distribution in an organism’s genome are attributed to the underlying neutral mutation processes they undergo (54, 56). Previous studies involving quantitative experiments have shown that polymerase-induced slippage rates are higher with the increase in the number of repeat units involved and are inversely correlated to the repeat unit length (54).

PCA (**Figure 3**), carried out using data obtained for both sets of markers, revealed the overall variability among populations, including the divergence between groups (i.e., structured genetic variability) and the variation occurring within groups (random genetic variability). The PCA results revealed variations between the BPH populations under study. Even though BPH populations from New Delhi, Ludhiana, Rajnagar, Aizawl and Warangal form a close cluster, they are still discernible from each other, thereby indicating the resolving power of the markers used in the analysis. Further, these results were also supported by the phylogenetic analysis (**Figure S2**), where a similar clustering pattern was observed. These results, while signifying the relatedness of the north, south and north-eastern BPH populations, also indicate the divergence from each other, thereby demonstrating the capacity of these markers to reveal polymorphisms capable of differentiating the populations. Further, the VIP scores plot obtained from PLS-DA helped identify and rank the markers according to their capacity to differentiate population clusters. Screening with both TR and SSR markers revealed high levels of genetic variability within the populations, possibly a consequence of the highly migratory nature of the insect. Despite the high intra-population variabilities, the markers developed in the current study could identify population-specific features.

In addition, we also detected a large number of SNPs in the sequences associated with the Control Region of the nine populations. Based on SNPs, the total nucleotide diversity observed for the nine BPH populations was as high as >5%, indicating that these insects could have undergone several possible admixture events due to their migratory nature. Based on the SNPs studied, three populations from the south (Hyderabad, Nalgonda and Warangal) showed a high level of relatedness. Interestingly, these populations also showed a similar level of relatedness to BPH populations from Ludhiana and Rajnagar. This suggests that the southern BPH population may be the source of the northern and north-eastern BPH populations due to migration, as it has been shown for aphids where its migration is primarily aided by the monsoon winds that are a strong driver of wind patterns in the Indian subcontinent (57–60). In India, the south-westerly winds commence after the summer months, and the north-eastern winds predominate in the autumn months and are prominent near the Indo-Gangetic region. These seasonal winds can also carry these insects over large distances and across several geographic barriers, thereby influencing the migration dynamics of BPH populations in India.

As discussed earlier, BPH populations are unlikely to overwinter in the northern and the north-eastern parts of the country. This, coupled with the unavailability of the rice host for BPH to feed on due to the lack of rice cultivation in the northern states of India during the winter months, is likely to induce a form switch in BPH to the macropterous form. Further, the north-easterly winds carry these insects to the southern region, where the weather, along with the availability of the host, provides the insects with a favorable environment for its growth and survival. However, during the summer monsoon, the wind direction reverses, and the south-westerly winds become active and likely act as a carrier of these insects from the southern states to the northern region, where the rice host is now available for the insects to feed on. In addition, except for the Bishnupur population, our data suggest no obvious evidence of isolation by distance (IBD) between the geographically distinct populations under study, as also shown in earlier studies (39, 42, 61). Geographically, the Purvanchal mountain range splits the north-eastern states (**Figure S1**). One of our collection sites (Bishnupur) is located east of this mountain range, while the Aizawl and Rajnagar collection sites are situated on the western side of this mountain range. Therefore, in all likelihood, this geographical barrier hinders the migration of insects from Bishnupur to Aizawl and Rajnagar and vice versa. Thus, the origin of planthopper populations in Bishnupur could be from areas further east of Bishnupur, i.e., from the rice-growing regions of the Indo-Chinese peninsula. Future studies involving BPH populations from these areas could help confirm this hypothesis.

To summarize, we show that the markers developed from the Control Region of the BPH mitogenome can reliably differentiate the BPH populations representing nine different rice-growing areas in India. And as stated earlier, these markers can be used to screen a larger set of populations, especially those from the Indo-China regions. In addition, and to the best of our knowledge, this is for the first time that markers developed from the Control Region of the BPH mitogenome are successfully used for differentiating BPH populations. The variable number of tandem repeats, the SNPs and varied restriction patterns obtained from individuals representing nine different BPH populations provided evidence of the likely migration dynamics and dispersal ability of BPH. Subsequently, this data can be used to devise suitable and science-based management strategies for this important pest of rice.

## Data availability statement

The datasets presented in this study can be found in online repositories. The names of the repository/repositories and accession number(s) can be found in the article/**Supplementary Material**.

## Author contributions

SN, YS and JB conceived the project. RA performed investigation, formal analysis, interpretation and curation, writing- original draft and editing. NS, SS, YS and JB contributed to the collection of reagents/materials. SN provided resources, performed project administration, investigation. provided biological samples. SM-L, YS, JB and SN provided supervision and writing-review and editing. The final manuscript was read and approved by all the authors.

## Funding

This research work was partially funded by a grant (BT/PR16912/NER/95/346/2015) from the Department of Biotechnology (DBT), Government of India, to YS, JB and SN. Research in SN’s laboratory is partially supported by core grants from ICGEB, extra-mural funding from the DBT and the Science and Engineering Research Board (SERB).

## Acknowledgments

SN thanks ICGEB for the award of the Arturo Falaschi Emeritus Scientist position. RA thanks DBT for a Junior Research Fellowship and the University of Delhi for a PhD fellowship (2018-2019).

## Conflict of interest

The authors declare that the research was conducted in the absence of any commercial or financial relationships that could be construed as a potential conflict of interest.

## Publisher’s note

All claims expressed in this article are solely those of the authors and do not necessarily represent those of their affiliated organizations, or those of the publisher, the editors and the reviewers. Any product that may be evaluated in this article, or claim that may be made by its manufacturer, is not guaranteed or endorsed by the publisher.

## Supplementary material

Supplementary Figure 1 Map indicating BPH collection sites in India. The Purvanchal range and the monsoon wind directions are indicated. The regions highlighted in red indicate sample collection sites. One collection site each from Delhi, Manipur, Mizoram, Odisha, Punjab and Tripura and three collection sites (districts) from Telangana are indicated. The brown triangles represent the Purvanchal range or the Eastern Himalayan range in the north-east states. The blue and orange arrows indicate the south-westerly (summer monsoon) and north-easterly (winter monsoon) winds, respectively.

Supplementary Figure 2 Phylogenetic tree inferred from the consensus sequences of the mitochondrial Control Region of the BPH populations used in the study (see Materials and Methods for details). The evolutionary relationship was inferred using the UPGMA method. The consensus tree was inferred from 1000 replications, and figures at the nodes represent bootstrap values above 50%. Branch lengths are arbitrary.
