# Supplementary figures and images for: Polymorphisms in the hypervariable control region of the mitochondrial DNA differentiate BPH populations
Source: Front Insect Sci. 2022 Nov 2;2:987718. doi: 10.3389/finsc.2022.987718 (PMC10926497; doi:10.3389/finsc.2022.987718)

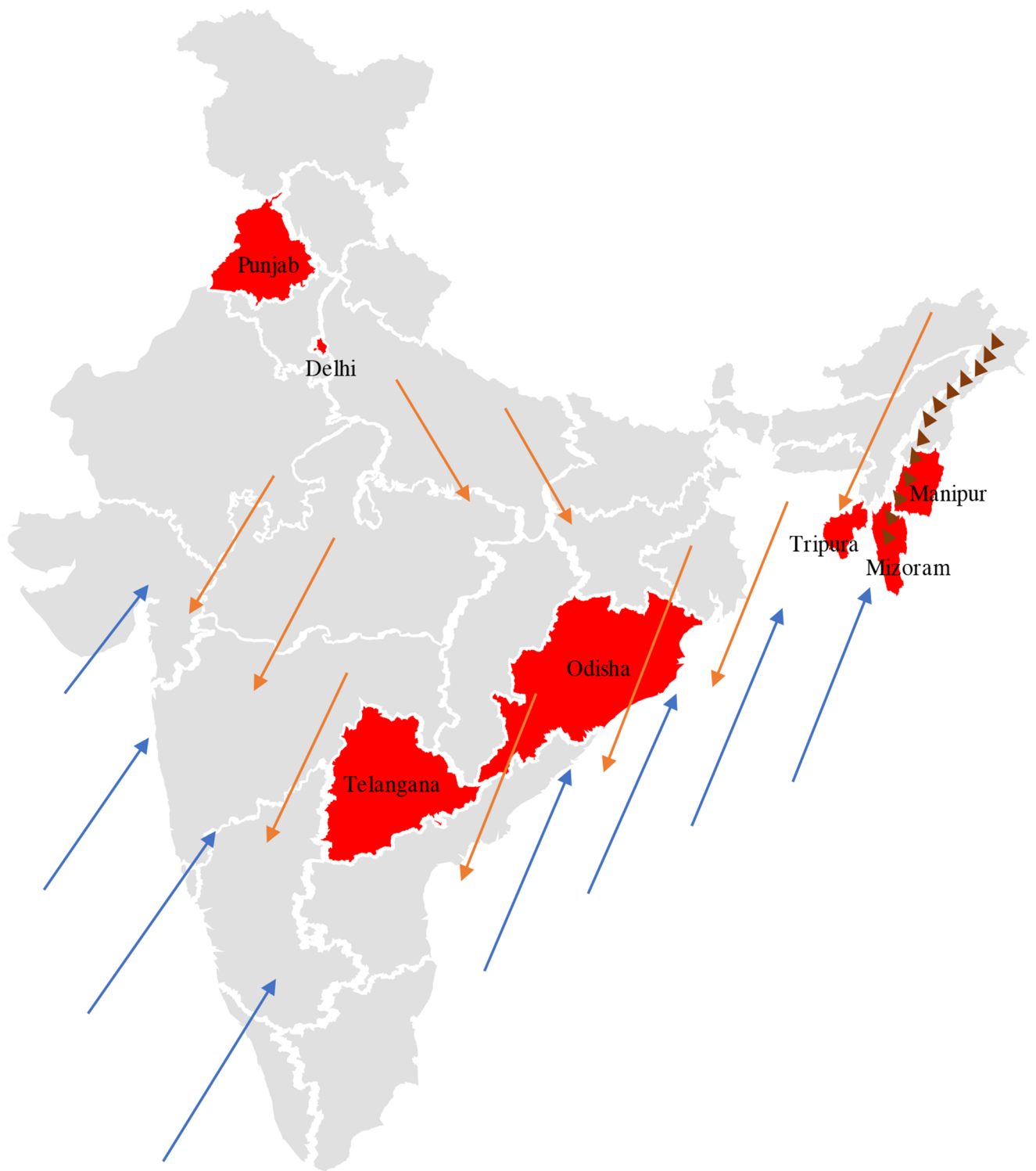

Supplement: Supplementary Figure 1 — Map indicating BPH collection sites in India. The Purvanchal range and the monsoon wind directions are indicated. The regions highlighted in red indicate sample collection sites. One collection site each from Delhi, Manipur, Mizoram, Odisha, Punjab and Tripura and three collection sites (districts) from Telangana are indicated. The brown triangles represent the Purvanchal range or the Eastern Himalayan range in the north-east states. The blue and orange arrows indicate the south-westerly (summer monsoon) and north-easterly (winter monsoon) winds, respectively. [file Image_1.pdf]

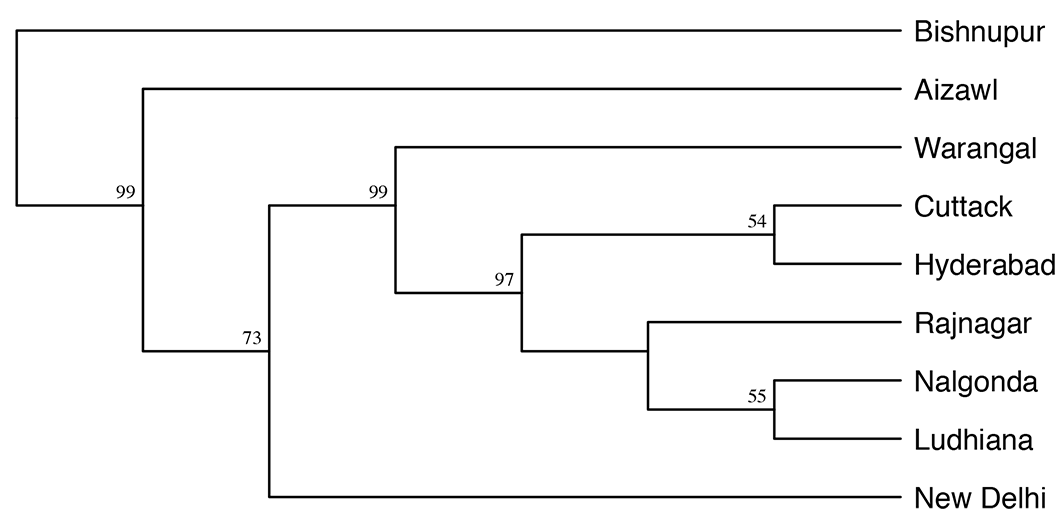

Supplement: Supplementary Figure 2 — Phylogenetic tree inferred from the consensus sequences of the mitochondrial Control Region of the BPH populations used in the study (see Materials and Methods for details). The evolutionary relationship was inferred using the UPGMA method. The consensus tree was inferred from 1000 replications, and figures at the nodes represent bootstrap values above 50%. Branch lengths are arbitrary. [file Image_2.tif]
